# The epidemiology and evolutionary dynamics of massive dengue outbreak in China, 2019

**DOI:** 10.3389/fmicb.2023.1156176

**Published:** 2023-04-17

**Authors:** Shaowei Sang, Yujuan Yue, Yiguan Wang, Xiangwei Zhang

**Affiliations:** ^1^Clinical Epidemiology Unit, Qilu Hospital of Shandong University, Jinan, Shandong, China; ^2^Clinical Research Center of Shandong University, Jinan, Shandong, China; ^3^State Key Laboratory of Infectious Disease Prevention and Control, Chinese Center for Disease Control and Prevention, National Institute for Communicable, Disease Control and Prevention, Beijing, China; ^4^Institute of Ecology and Evolution, University of Edinburgh, Edinburgh, United Kingdom; ^5^Department of Thoracic Surgery, Shandong Provincial Hospital Affiliated to Shandong First Medical University, Jinan, Shandong, China

**Keywords:** dengue, epidemiology, imported, China, evolution

## Abstract

**Introduction:**

In 2019, China experienced massive dengue outbreaks with high incidence and expanded outbreak areas. The study aims to depict dengue’s epidemiology and evolutionary dynamics in China and explore the possible origin of these outbreaks.

**Methods:**

Records of confirmed dengue cases in 2019 were obtained from the China Notifiable Disease Surveillance System. The sequences of complete envelope gene detected from the outbreak provinces in China in 2019 were retrieved from GenBank. Maximum Likelihood trees were constructed to genotype the viruses. The median-joining network was used to visualize fine-scale genetic relationships. Four methods were used to estimate the selective pressure.

**Results:**

A total of 22,688 dengue cases were reported, 71.4% of which were indigenous cases and 28.6% were imported cases (including from abroad and from other domestic provinces). The abroad cases were predominantly imported from Southeast Asia countries (94.6%), with Cambodia (3,234 cases, 58.9%), and Myanmar (1,097 cases, 20.0%) ranked as the top two. A total of 11 provinces with dengue outbreaks were identified in the central-south of China, of which Yunnan and Guangdong provinces had the highest number of imported and indigenous cases. The primary source of imported cases in Yunnan was from Myanmar, while in the other ten provinces, the majority of imported cases were from Cambodia. Guangdong, Yunnan and Guangxi provinces were China’s primary sources of domestically imported cases. Phylogenetic analysis of the viruses in outbreak provinces revealed three genotypes: (I, IV, and V) in DENV 1, Cosmopolitan and Asian I genotypes in DENV 2, and two genotypes (I and III) in DENV 3. Some genotypes concurrently circulated in different outbreak provinces. Most of the viruses were clustered with those from Southeast Asia. Haplotype network analysis showed that Southeast Asia, possibly Cambodia and Thailand, was the respective origin of the viruses in clade 1 and 4 for DENV 1. Positive selection was detected at codon 386 in clade 1.

**Conclusion:**

Dengue importation from abroad, especially from Southeast Asia, resulted in the dengue epidemic in China in 2019. Domestic transmission between provinces and positive selection on virus evolution may contribute to the massive dengue outbreaks.

## Introduction

Dengue is a mosquito-borne viral disease caused by four antigenically distant serotypes (DENV 1-4), and each serotype can be classified into distinct genotypes based on the similarity of complete envelope (E) gene (Chen and Vasilakis, 2011). Dengue is endemic in many tropical and subtropical populated areas, including Southeast Asia, South Asia, the Americas, and the Western Pacific (Guzman and Harris, 2015). As dengue becomes more prevalent in these hyper-endemic regions (Zambrano et al., 2019), it frequently spreads to regions that were previously free of dengue (Smith et al., 2005; Wilder-Smith et al., 2014; Succo et al., 2016; Kobayashi et al., 2018). Globally, the burden of dengue has significantly increased since 1990, with dengue cases rising by 85.5% (Yang et al., 2021).

Although dengue is still considered an imported disease in China (Sang et al., 2015a,^2021^; Sun et al., 2020), the incidence has increased dramatically with expanded outbreak regions in recent years. The first dengue outbreak was reported in 1978 in Guangdong Province, Southeast China. And then, the dengue gradually expanded from the Southeast coastal region to central China (Huang et al., 2014; Yao et al., 2020) and to the Western region (Zhang et al., 2014).

The year 2019 is one of the years most affected by dengue, and many countries have experienced intense dengue activity (Zambrano et al., 2019; Mwanyika et al., 2021; Poudyal et al., 2021). The Americas Region reported the highest number of cases since 1980 (Zambrano et al., 2019). Massive outbreaks occurred in Nepal (Poudyal et al., 2021) and Tanzania (Mwanyika et al., 2021). Bhutan also experienced the first nationwide dengue epidemic (Tsheten et al., 2021). In China, dengue was highly active in 2019, with the reported cases reaching the second highest number between 2005 and 2020. Additionally, the number of provinces affected by the disease significantly increased (Yue et al., 2022). The southern province of Hainan reported its first dengue outbreak after disappearing for over 20 years (Du et al., 2021). Dengue viruses are diverse, but there is no systematic study analyzing the genetic diversity of the viruses from outbreak provinces. Positive selection, a driving force of virus evolution, plays an important role in the genetic variation of DENV. However, little is known about positive selection sites of the circulating dengue viruses in China in 2019. In this study, we systematically leverage dengue case data and viral sequence data collected in China in 2019 to characterize the epidemiology and evolutionary dynamics of dengue outbreak and its possible origins.

## Materials and methods

### Data collection

The dengue data were downloaded from China Notifiable Disease Surveillance System covering 31 (91.2%) (Hong Kong, Macau, Taiwan not included) provincial-level administrative regions (Supplementary Appendix 1). Dengue cases are diagnosed according to the China National diagnostic criteria for dengue fever (WS216-2008), enacted by the National Health Commission of China referring to WHO diagnostics criteria in consideration of China conditions. In brief, dengue cases were categorized as clinically diagnosed and laboratory-confirmed cases. Clinically diagnosed cases were identified by experienced local physicians according to the clinical manifestations (e.g., acute onset of rash, headache, fever, itching, anorexia, or arthralgia), epidemiologic exposure history and laboratory test (reduction in white blood-cell counts and/or platelet count, positive in IgM or NS1). Laboratory-confirmed cases were determined based on clinically diagnosed cases presenting with any of the following lab test results relating to dengue: a four-fold increase in specific IgG antibody titer, positive on a PCR test or virus isolation and identification test. Records of confirmed dengue cases, including date of onset, the reporting province, and whether indigenous or imported (with source region), were used to conduct the epidemiological analysis.

The imported dengue case is defined as the patient who had a traveling history to a dengue-affected area and reportedly being bitten by mosquitoes within 15 days before the onset of illness; or defined based on laboratory results showing that the detected dengue virus had a high sequence similarity to viruses isolated from the putative source region where the patient had traveled to. Otherwise, the dengue case is considered to be an indigenous case (Li et al., 2012). In the study, the imported cases are further categorized into cases imported from abroad and cases from other domestic provinces.

The complete E gene sequence data of the virus from the outbreak provinces in China in 2019 were retrieved from GenBank. The search terms included “dengue,” “China” and “2019.” The complete E gene sequences that could not be determined on their outbreak provinces by checking location in sequence information or published papers were excluded. We chose only one sequence to represent sequences with 100% similarity in the same outbreak province (Supplementary Appendix 2). These included sequences were then compared with published sequences (as of March 9th 2022) by using the nucleotide blast program in the NCBI. We chose one sequence if the references had identical similarities in the same location and year. All the references with complete E gene were downloaded with the accession number, collection date, and country (Supplementary Appendix 3).

### Epidemiological analysis

ArcGIS 10.1 (ESRI, Redlands, CA, USA) was used to visualize the spatial distribution of all the dengue cases from China Notifiable Disease Surveillance System. The cases identified in China were aggregated at the provincial level, while the cases introduced from Southeast Asia were aggregated at the national level. A time series of dengue cases was visualized using ggplot2 package in R 4.1.0. A pyramid plot on dengue cases in outbreak provinces was conducted using Plot Pyramid function in DescTools package. A Sankey plot was conducted using sankeyNetwork function in networkD3 package to visualize the mobility of dengue cases across provinces or countries (Supplementary Appendix 4).

### Genotyping

All the sequences were aligned using MAFFT (Katoh et al., 2009). Maximum Likelihood (ML) trees for each serotype were constructed using PhyML 3.0 (Guindon et al., 2010), incorporating a GTR + I + Γ4 model (general time-reversible model with a proportion of invariable sites and four gamma-distributed rate categories). The reliability of branching pattern was tested through 1,000 bootstrap sampling. In order to better describe the molecular epidemiology of dengue virus in outbreak provinces, the ML trees were labeled with different clades using the definition of a minimum of three sequences of monophyletic origin. Haplotype network analysis and selective pressure analysis were further conducted in clades with viruses from more than three outbreak provinces. The ggtreeExtra package in R 4.1.0 was used for visualization and annotation of ML trees (Xu et al., 2021; Supplementary Appendix 4).

### Haplotype network analysis

The median-joining network (Bandelt et al., 1999) was used to visualize fine-scale genetic relationships based on single nucleotide polymorphisms in analyzed sequences, which was analyzed using DnaSP 6.12.3 (Rozas et al., 2017) and PopART 1.7 (Leigh and Bryant, 2015). The similar haplotypes sharing a common ancestor were separated into different haplogroups.

### Estimating selective pressure

The underlying selection pressures were measured using the Datamonkey web-server.^1^ The positively selected sites were detected by four different methods, including mixed effects model of evolution (MEME) (Murrell et al., 2012), fixed effects likelihood (FEL) (Kosakovsky Pond and Frost, 2005), fast, unconstrained Bayesian approximation (FUBAR) (Murrell et al., 2013), and single-likelihood ancestor counting (SLAC) (Kosakovsky Pond and Frost, 2005). In brief, FEL, SLAC and FUBAR assumes that the selection pressure for each site is constant, and, respectively use a ML approach, a combination of ML and counting approaches, and a Bayesian approach to infer non-synonymous (*d*_*N*_) to synonymous (*d*_*S*_) substitution rates on a per-site. MEME employs a mixed-effects ML approach to test the hypothesis that individual sites have been subject to episodic positive or diversifying selection. In the cases of FEL, SLAC, and MEME, codons with *p*-value < 0.1 suggest regions under positive selection, and for FUBAR posterior probability >0.9 was considered positive. Sites were considered under positive selection if supported by at least two methods.

### Ethics statement

Ethical approval for the study was obtained from Qilu Hospital of Shandong University Ethical Committee (No. KYLL-2016(KS)-031). The epidemiology data and genetic data were retrospectively obtained from China Notifiable Disease Surveillance System and GenBank, respectively, and the privacy of the patient was not included in the data. Therefore, the informed consent was exempted.

## Results

### Dengue cases in China

**FIGURE 1:**
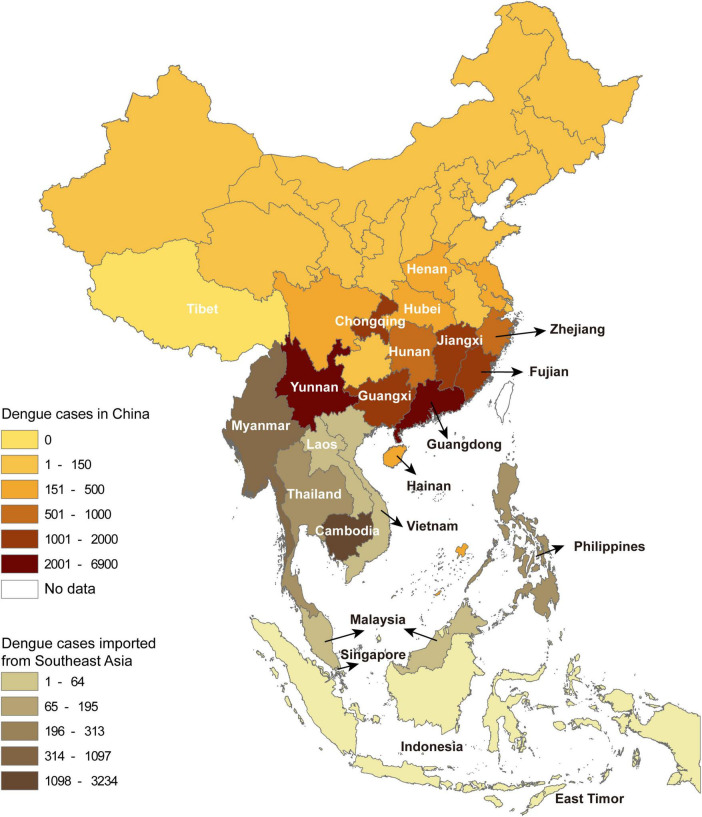
The spatial distribution of dengue cases in China by province and the cases introduced from Southeast Asia by country.

A total of 22,688 confirmed dengue cases were recorded in China Notifiable Disease Surveillance System in 2019. Indigenous cases and imported cases (including from abroad and domestic provinces) accounted for 71.4 and 28.6%, respectively, covering all the studied provinces except Tibet. The number of reported dengue cases by province gradually decreased from the south to the north. A total of 11 provinces with dengue outbreaks were identified in the central-south of China, including Henan, Hubei, Chongqing, Hunan, Jiangxi, Zhejiang, Fujian, Guangdong, Guangxi, Yunnan, and Hainan (Figure 1).

The dengue cases from abroad were predominantly imported from Southeast Asia (5,492), followed by South Asia (213), Africa (53), the Americas (17), the Middle East (17), and Oceania (7). Among the imported cases from Southeast Asia, 58.9% (3,234) came from Cambodia, followed by Myanmar (20.0%, 1,097) (Figure 1).

**FIGURE 2:**
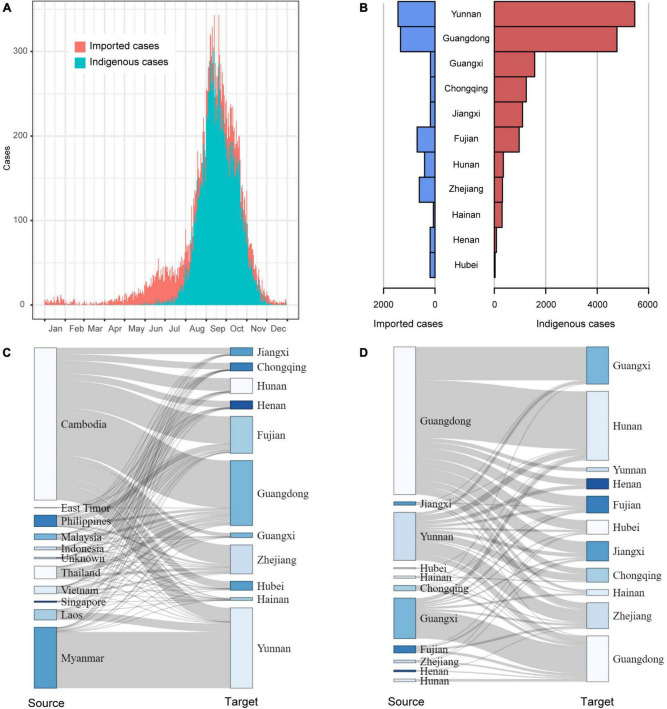
The field epidemiology of dengue cases in China in 2019. **(A)** The time series of dengue cases; **(B)** The dengue cases in outbreak provinces; **(C)** The spatial distribution of Southeast Asia dengue cases introduced to outbreak provinces; **(D)** The mobility of domestic dengue cases among outbreak provinces.

Temporally, dengue cases were reported all year round, but 79.6% of them occurred in August (4,028), September (8,335), and October (5,706). There were more imported cases than indigenous cases from January to July and in December. The indigenous cases gradually appeared in June, peaked in September, then sharply decreased in November, and finally ended with a few sporadic cases in December (Figure 2A).

### Dengue outbreaks in China

Of the 11 provinces with dengue outbreaks in 2019, Yunnan and Guangdong provinces had the highest number of imported and indigenous cases (Figure 2B). All the 11 provinces (except Hunan, Zhejiang, Henan and Hubei) reported more indigenous cases than imported cases. A majority of the imported cases were from Cambodia and then Myanmar. As Yunnan Province shares a long border with Myanmar, it isn’t surprising that most of the imported cases in Yunnan came from Myanmar, followed by Cambodia and Laos. For the other ten provinces, Cambodia was the primary source of imported cases (Figure 2C). In addition, Fujian Province also had a proportion of imported cases from the Philippines, and Guangdong Province had some from Malaysia, Thailand, and Vietnam.

Domestically, Guangdong, Yunnan and Guangxi provinces were the primary sources of imported cases (Figure 2D). In particular, cases from Guangdong had been introduced to all the other outbreak provinces, especially adjacent Hunan and Guangxi provinces.

### Molecular epidemiology of dengue viruses from outbreak provinces in China

**FIGURE 3:**
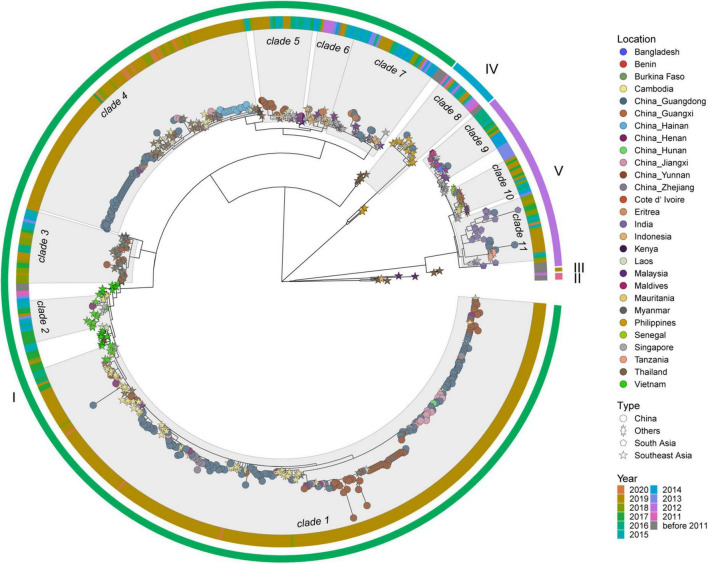
The ML tree of DENV 1. The three layers annotating the tree from outside to inside are: the genotype, year of isolation and location.

DENV 1 ML tree was constructed using 631 strains from outbreak provinces in China in 2019 (303 strains) and reference strains (328 strains) (Figure 3 and Supplementary Appendix 5). Three genotypes (I, IV and V) were detected in these provinces, with genotype I in Guangdong, Guangxi, Hainan, Henan, Hunan, Jiangxi, Yunnan and Zhejiang provinces, genotype IV and genotype V in Guangdong Province. Genotype I and IV clustered with viruses from Southeast Asia (Cambodia, Vietnam, Thailand, Singapore, Myanmar, Laos, Malaysia, Indonesia and the Philippines), while Genotype V clustered with viruses from Southeast Asia (mainly Singapore, Malaysia and Thailand), South Asia (mainly Maldives, Bangladesh and India), and Africa (mainly Burkina Faso, Senegal, Benin, Cote d’ Ivoire, Mauritania, Eritrea, Kenya and Tanzania) (Supplementary Appendix 6).

Genetic variation of dengue viruses from clade 1 was analyzed and revealed seven haplotype groups (Supplementary Appendix 7). Viruses from Vietnam (2016–2017) were identified at the bottom of clade 1 (Supplementary Appendix 7A), and were the most recent common ancestor of clade 1. These viruses from Vietnam corresponded to the Haplogroup 1 in the haplotype network analysis (Supplementary Appendix 7B). Haplogroup 2, as the offspring of Haplogroup 1, mainly included the viruses from Cambodia. Haplogroups 3-7 were the offspring of haplogroup 2. Some viruses from Cambodia having identical sequences with some viruses from China were the core of haplogroups 3-7. The positive selection analysis based on the dataset of clade 1 identified one site (codon 386) under positive selection indicated by MEME, FEL, and FUBAR methods (Supplementary Appendix 8).

Genetic variation analysis shows that viruses from China in clade 4 separately clustered with viruses from Southeast Asia in 2017–2020 (Supplementary Appendix 9A). The haplotype network analysis showed that viruses in clade 4 were separated into three haplogroups (Supplementary Appendix 9B), with viruses from Southeast Asia, mainly from Thailand, being the core. Haplogroups 2 and 3 were the offspring of haplogroup 1, and some viruses from Thailand having identical sequences with some viruses from China were the core of haplogroups 2 and 3. No sites were identified under positive selection in clade 4 (Supplementary Appendix 8).

**FIGURE 4:**
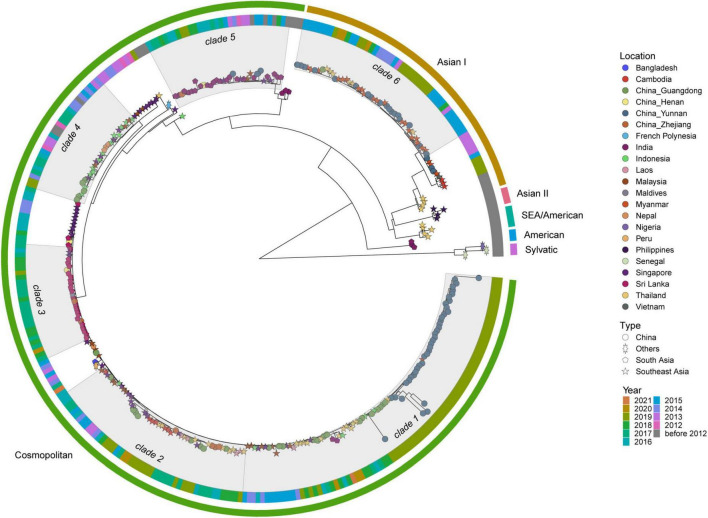
The ML tree of DENV 2. The three layers annotating the tree from outside to inside are: the genotype, year of isolation and location.

DENV 2 ML tree was constructed using 333 strains, including 63 strains from outbreak provinces and 270 reference strains (Figure 4 and Supplementary Appendix 10). Two genotypes, Cosmopolitan and Asian I genotypes, were identified in the outbreak provinces, with Cosmopolitan genotype in Yunnan, Guangdong and Henan provinces, and Asian I genotype in Guangdong and Yunnan provinces. Cosmopolitan genotype detected in outbreak provinces were mainly clustered with viruses from Southeast Asia (Myanmar, Thailand, Indonesia, Singapore, Malaysia, Laos, Cambodia) and South Asia (India, Sri Lanka, Nepal). In contrast, Asian I genotype only clustered with viruses from Southeast Asia (mainly Myanmar and Thailand) (Supplementary Appendix 6).

**FIGURE 5:**
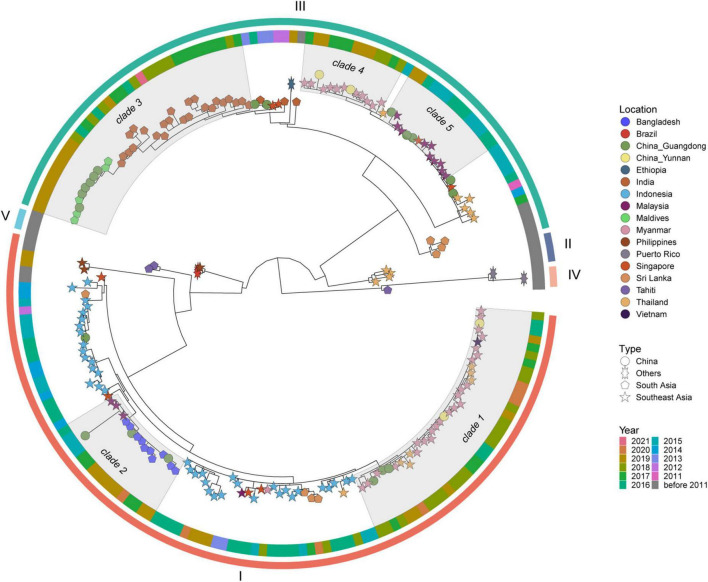
The ML tree of DENV 3. The three layers annotating the tree from outside to inside are: the genotype, year of isolation and location.

Figure 5 shows the DENV 3 ML tree constructed using 199 strains from outbreak provinces (12 strains) and reference strains (187 strains) (Supplementary Appendix 11). Two genotypes, genotype I and III, were detected in outbreak provinces, with genotype I in Guangdong Province and genotype III in Guangdong and Yunnan provinces. Genotype I and III viruses detected in outbreak provinces were mainly clustered with viruses from Southeast Asia (mainly Myanmar, Vietnam, Thailand, Malaysia, and Singapore) and South Asia (mainly Bangladesh, India, and Maldives) (Supplementary Appendix 6).

## Discussion

Dengue is an emerging global health concern requiring more attention from both dengue endemic and non-endemic regions. In 2019, dengue continued to be widespread in China, with the greatest number of provinces reporting outbreaks of the disease. Southeast Asian countries, especially Cambodia and Myanmar, were China’s primary source of imported cases. Phylogenetic analysis showed that multiple genotypes cocirculated in outbreak provinces, and some genotypes concurrently circulated in different outbreak provinces.

Southeast Asia is usually the epicenter of dengue worldwide. A systematic review showed that the majority of imported dengue cases worldwide were from Southeast Asia (Gwee et al., 2021). China is adjacent to Southeast Asia, and the two regions have frequent travel and commercial trade. The dengue surveillance data between 2013 and 2016 from China showed that imported cases occurred almost year-round, most of which came from Southeast Asia (Sang et al., 2021). The surveillance data from China in 2019 showed that most of the imported cases were from Cambodia and Myanmar. Dengue is endemic in Cambodia. The Cambodia Ministry of Health has been monitoring the incidence of dengue every week since the 1995 outbreak. The most recent dengue epidemics in Cambodia were in 2007 (39,618 cases) and 2012 (42,362 cases). However, Cambodia reported a dramatic increase in 2019, with cases of 68,597 (Cousien et al., 2019; Boyer et al., 2022). In 2019, Chinese business people set up new factories in Cambodia and organized a large number of domestic workers to work there, which may have resulted in an increase of imported dengue cases from Cambodia. Dengue is also endemic in Myanmar. Both Myanmar and Cambodia were classified as high-burden dengue countries in the Asian Pacific region (Shepard et al., 2013). The Myanmar Ministry of Health and Sport data showed that the dengue situation was still severe in 2019, with reported cases of over 23,000 (NewsDesk, 2019). Yunnan Province is located in southwest China, bordering Myanmar. Our study showed that Myanmar cases were predominantly imported to Yunnan Province, consistent with the surveillance data from 2004 to 2018 in China (Yue et al., 2021). In line with these observations, the phylogenetic analysis indicated that Southeast Asia was China’s major source of imported cases in 2019. Therefore, the results from the field investigation data and genetic data support the conclusion that dengue in Southeast Asia played a crucial role in the massive dengue outbreaks in China. This role was further demonstrated during the pandemic of Coronavirus Disease 2019 (Jiang et al., 2021; Xiao et al., 2021). Because of the strict border restrictions and quarantine policies, the average annual number of imported dengue cases dropped by 88.7%, and the total dengue cases consequently decreased by 98.1% in 2020 in Guangdong Province (Xiao et al., 2021). All the above suggested that dengue was still an imported disease in China (Jiang et al., 2021). Close surveillance of imported cases could be a low-cost, effective way to alert dengue outbreaks (Sang et al., 2015b; Massad et al., 2018).

A total of 11 provinces reported dengue outbreaks in China in 2019, which was also attributed to the widely distributed mosquito besides a mass of imported cases from abroad. *Aedes aegypti* and *Aedes albopictus* are the primary and secondary vectors of dengue globally. In China, *A. albopictus* is widely distributed in nearly half of China from northeast to southwest, while *A. aegypti* thrives only in some areas of Guangdong, Guangxi, Yunnan and Hainan provinces in Southern China and coexists with *A. albopictus* in these areas (Yue et al., 2022). The widely distributed *Aedes* mosquitoes provide the requisite for dengue outbreaks. Over half of the Chinese population live in the regions of dengue risk. Meanwhile, with population growth and the expansion of mosquito-inhabited areas, more people in China will be exposed to the risk of dengue (Kamal et al., 2018).

Weather condition is another vital factor explaining why dengue is an imported disease and why its prevalence has gradual decrease from the south to north in China. Temperature can affect dengue prevalence through multiple mechanisms, including influencing the *Aedes* mosquito biting rate, egg and immature mosquito development, extrinsic incubation period and survival at all stages of the mosquito life cycle (Ebi and Nealon, 2016). The winter temperature in most parts of China is relatively low, and mosquito densities sharply decrease in winter. Consequently, dengue virus transmissions are terminated in winter. However, dengue viruses will be re-introduced and spread as *Aedes* mosquitoes become more abundant and active under warmer temperatures in the next epidemic season, which may lead to an outbreak. That is also why indigenous cases appear in summer and autumn but only imported cases in cold seasons.

Repeated introductions of dengue cases from abroad are the main driver of dengue outbreaks in China (Sang et al., 2015a,^2021^; Sun et al., 2020). But would the domestic imported cases be another driver? Field investigation revealed the movement of the domestic case between outbreak provinces and the concurrent abroad cases imported to outbreak provinces (Figures 2C, D). Genetic data showed that dengue viruses from different provinces and abroad tended to be clustered together, with some even having identical sequences in some clades. Therefore, we speculated that imported cases from abroad and cases of interprovincial transmission possibly concurrently contributed to the prevalence of dengue in different provinces in China in 2019.

Dengue E protein consists of three structurally distinct domains: a central domain (EDI), a dimerization domain (EDII) and an immunoglobulin-like C terminal domain (EDIII) (Modis et al., 2004). EDIII spanning amino acid residues 300–400 of C-terminal of E protein is exposed and accessible on the virion surface. This domain functions in host cell surface receptor recognition and contains multiple type- and subtype-specific conformation-dependent neutralizing epitopes (Kuhn et al., 2002). Neutralizing epitopes of the dengue EDIII have been identified and included special residues 307, 333–351, and 383–389 (Modis et al., 2005). Mutations in these sites lead to escape from neutralization by conventional monoclonal neutralizing antibodies (Wu et al., 2003). The positive selection analysis showed amino acid residue 386 under strong positive selection, which may partly explain the massive dengue outbreak in China in 2019.

Although our study describes the epidemiology of dengue in China in 2019 by integrating field investigation data and genetic data, the results should be interpreted cautiously, given some limitations. First, the information on imported cases in the study was derived from the China Notifiable Disease Surveillance System. As dengue is a self-limited disease, some patients with no or mild symptoms might not seek medical attention and thus not be included in the system. Second, the dengue sequences from outbreak provinces in China in 2019 were retrieved from GenBank. It should be noticed that not all outbreak provinces (Chongqing, Fujian and Hubei provinces) deposited dengue sequences to GenBank. Besides, the local CDC in China conducted convenience sampling to genotype the viruses during the dengue epidemics. Since there was no reference for the sample size, the sampling cannot sufficiently reflect the diversity of dengue virus in China, and the international or domestic dengue transmission. Third, the inference on the possible origin of dengue viruses in China was based on the similarity of nucleotides to the strains from other countries. The strain sequences from some countries with limited resources for virologic diagnostic and reporting capacity may not be deposited into GenBank. For this consideration, we explored the origin of dengue viruses in a larger unit of world region instead of a specific country, which may mitigate the potential bias.

## Conclusion

Dengue is increasingly getting worse in the southern Chinese provinces, particularly Guangdong Province which is recognized as the most affected region and a primary source of domestically imported dengue cases (Wu et al., 2022), but the surveillance data from field investigation and genetic sequences consistently showed that viruses from abroad, especially from Southeast Asia, contributed to the dengue outbreak in China. Interprovincial mobility of domestic cases and importation from abroad resulted in large epidemics in different provinces. Our results support the development of an early warning system based on the imported cases within the region, in collaboration with bordering countries. Strengthening the surveillance and management of imported cases could be an efficient way to alleviate the burden of dengue in China.

## Data availability statement

The original contributions presented in this study are included in the article/Supplementary material, further inquiries can be directed to the corresponding authors.

## Ethics statement

The studies involving human participants were reviewed and approved by the Qilu Hospital of Shandong University Ethical Committee. Written informed consent for participation was not required for this study in accordance with the national legislation and the institutional requirements.

## Author contributions

SS and XZ: conceptualization and supervision. SS and YY: data curation. SS: formal analysis and funding acquisition. SS and YW: roles/writing–original draft. SS, YW, and XZ: writing–review and editing. All authors contributed to the article and approved the submitted version.

## Footnotes

1
www.datamonkey.org

## Supplementary material

Supplementary Appendix 1 Dengue cases downloaded from China Notifiable Disease Surveillance System.

Supplementary Appendix 2 The dengue viruses detected in outbreak provinces of China in 2019.

Supplementary Appendix 3 The reference strains in DENV 1-3.

Supplementary Appendix 4 The scripts for plot in R.

Supplementary Appendix 5 The ML tree of DENV 1.

Supplementary Appendix 6 Molecular epidemiology of dengue viruses in outbreak provinces of China.

Supplementary Appendix 7 The genetic variation of dengue viruses in clade 1 in DENV 1. A, the distribution of single-nucleotide polymorphisms; B, the result of haplotype network analysis. Each circle represents a unique haplotype. Color represents country or province of genome origin, and different colors in one circle mean that the sequences from different countries or provinces are identical. The size of the circle is proportional to number of viral sequences included. The black dot indicating the unsampled viruses.

Supplementary Appendix 8 Positive selection pressure analysis.

Supplementary Appendix 9 The genetic variation of dengue viruses in clade 4 in DENV 1. A, the distribution of single-nucleotide polymorphisms; B, the result of haplotype network analysis. Each circle represents a unique haplotype. Color represents country or province of genome origin, and different colors in one circle mean that the sequences from different countries or provinces are identical. The size of the circle is proportional to number of viral genomes included. T the black dot indicating the unsampled viruses.

Supplementary Appendix 10 The ML tree of DENV 2.

Supplementary Appendix 11 The ML tree of DENV 3.
