# Supplementary figures and images for: The epidemiology and evolutionary dynamics of massive dengue outbreak in China, 2019
Source: Front Microbiol. 2023 Apr 17;14:1156176. doi: 10.3389/fmicb.2023.1156176 (PMC10149964; doi:10.3389/fmicb.2023.1156176)

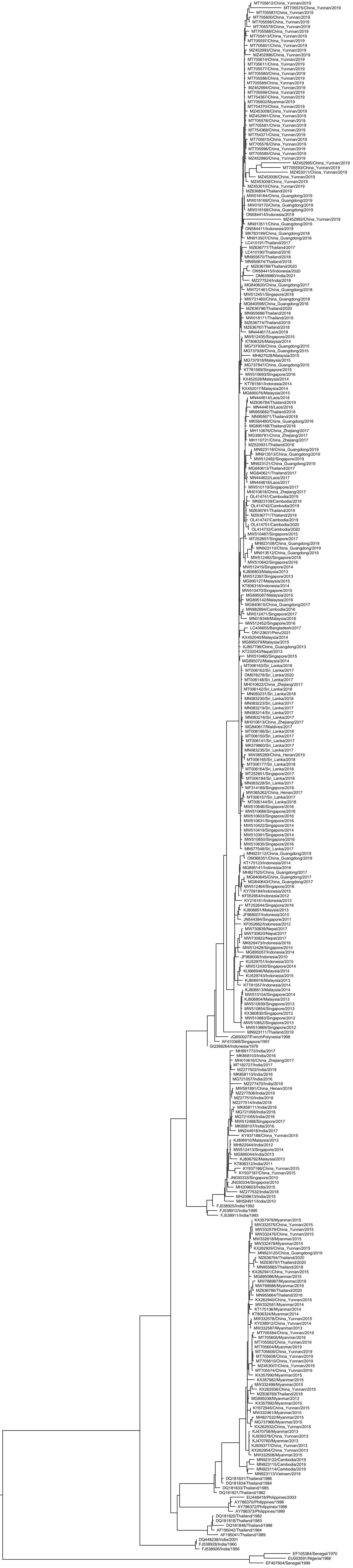

Supplement: Supplementary Appendix 1 — Dengue cases downloaded from China Notifiable Disease Surveillance System. [file Data_Sheet_1.ZIP › Supp/Appendix 10_DENV2_ML.pdf]

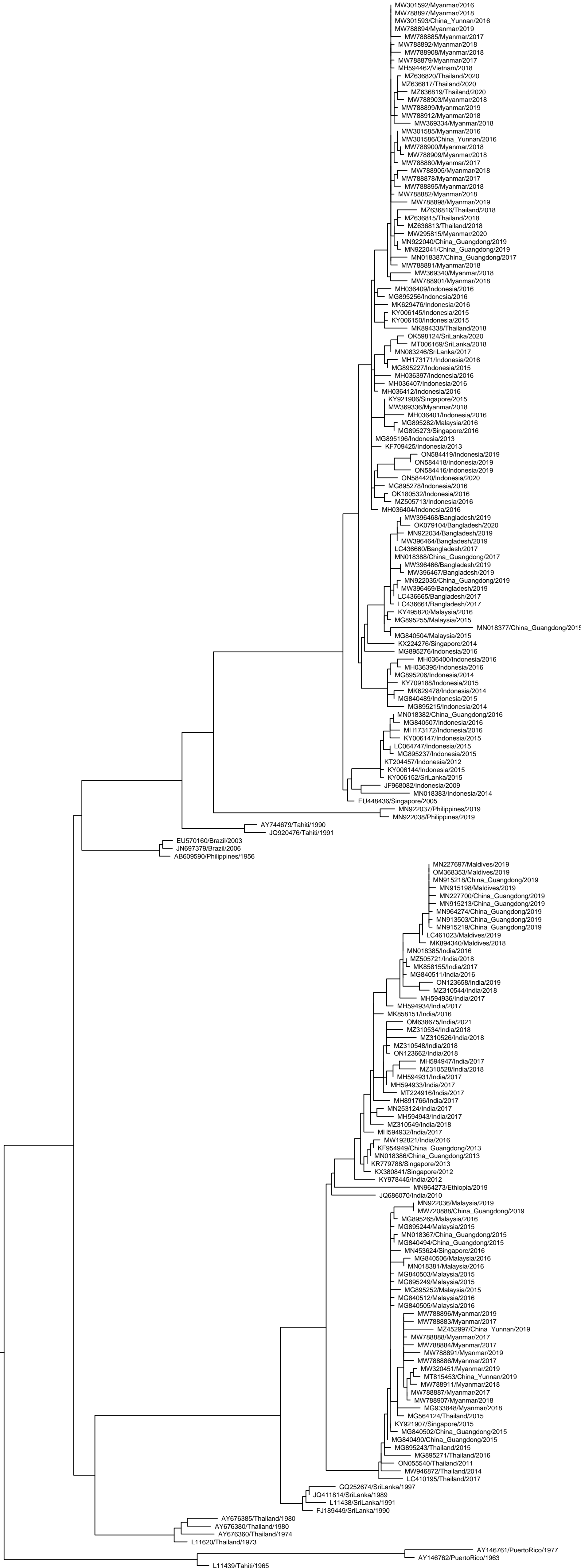

Supplement: Supplementary Appendix 1 — Dengue cases downloaded from China Notifiable Disease Surveillance System. [file Data_Sheet_1.ZIP › Supp/Appendix 11_DENV3_ML.pdf]

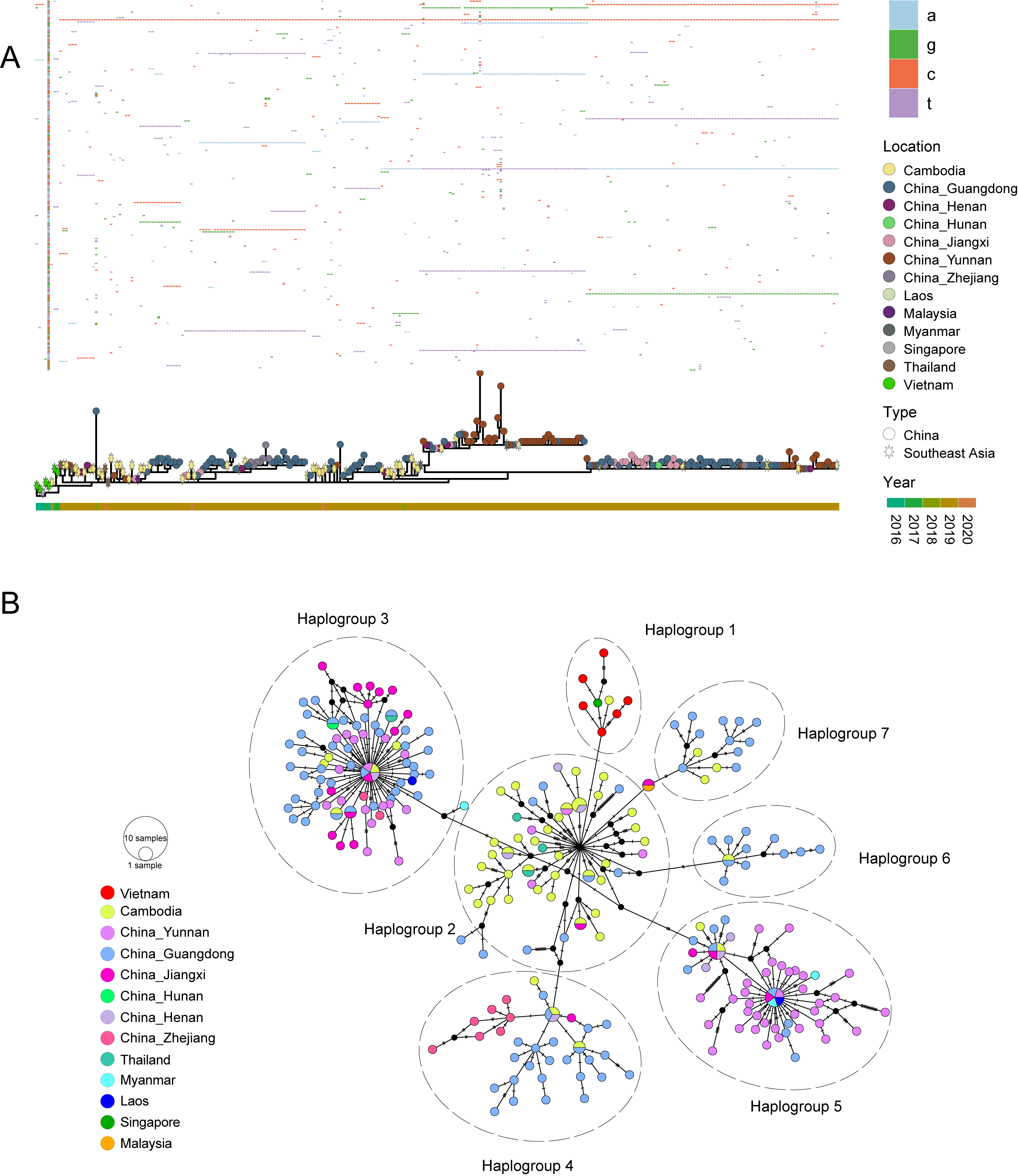

Supplement: Supplementary Appendix 1 — Dengue cases downloaded from China Notifiable Disease Surveillance System. [file Data_Sheet_1.ZIP › Supp/Appendix 7_clade1.tif]

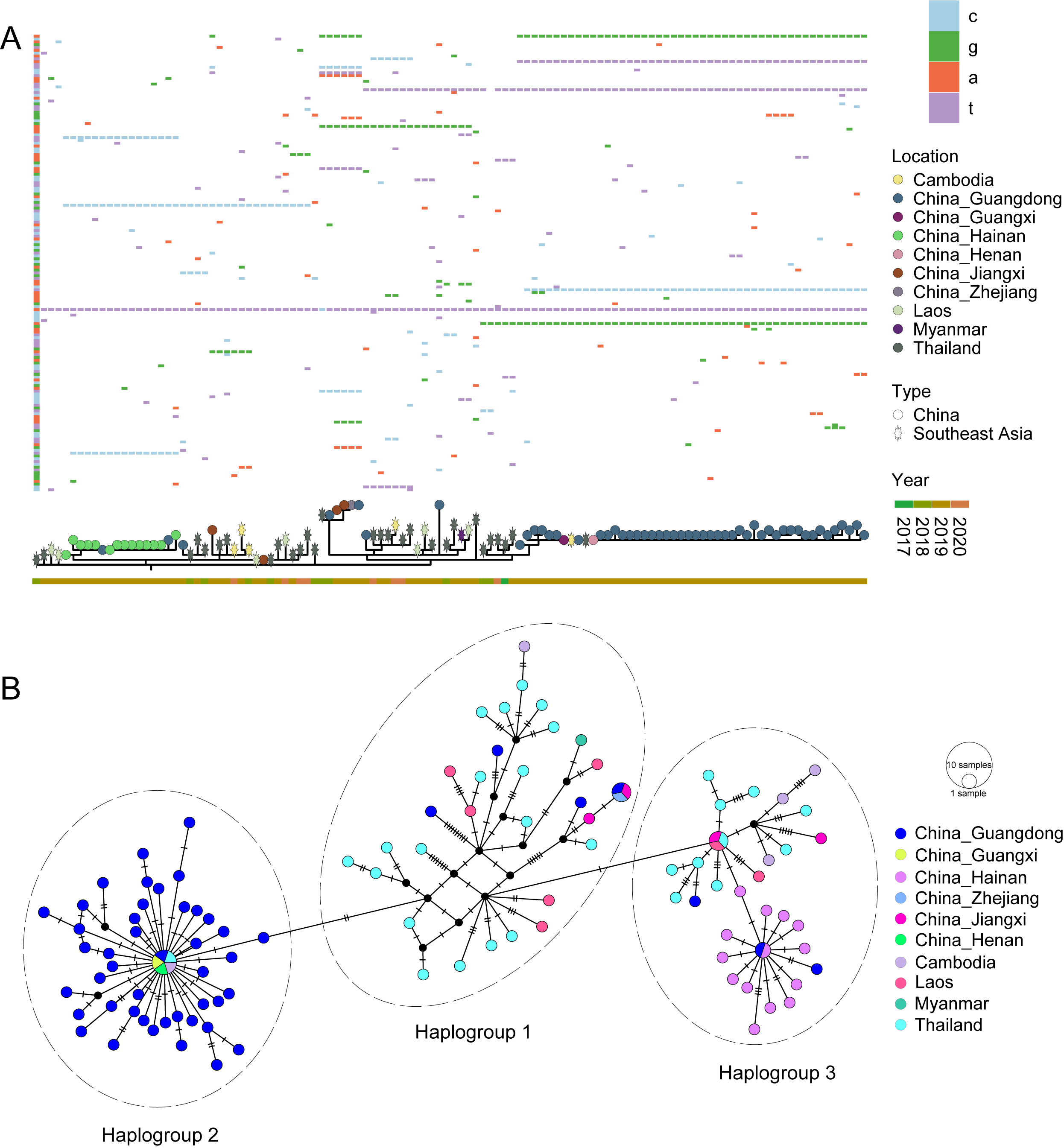

Supplement: Supplementary Appendix 1 — Dengue cases downloaded from China Notifiable Disease Surveillance System. [file Data_Sheet_1.ZIP › Supp/Appendix 9_clade4.tif]
