# Supplementary material for: The epidemiology and evolutionary dynamics of massive dengue outbreak in China, 2019
Source: Front Microbiol. 2023 Apr 17;14:1156176. doi: 10.3389/fmicb.2023.1156176 (PMC10149964; doi:10.3389/fmicb.2023.1156176)

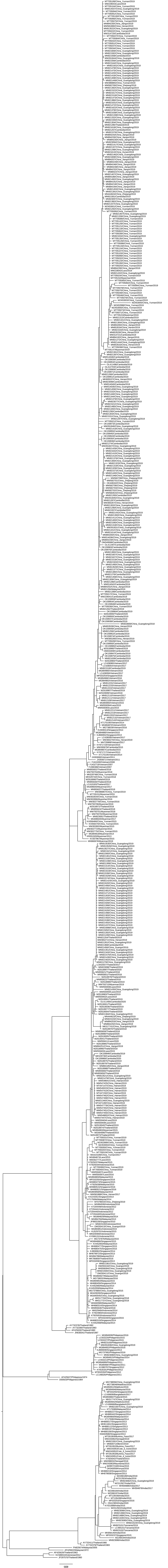

Supplement: Supplementary Appendix 1 — Dengue cases downloaded from China Notifiable Disease Surveillance System. [file Appendix 5_DENV1_ML.pdf › Supp/Appendix 5_DENV1_ML.pdf]
